# Synovial fluid leukocytes as diagnostic marker in periprosthetic shoulder infection

**DOI:** 10.1016/j.jseint.2024.09.011

**Published:** 2024-09-26

**Authors:** Stefan Köppe, Daniel Karczewski, Rony-Orijit Dey Hazra, Alp Paksoy, Agahan Hayta, Doruk Akgün

**Affiliations:** Department of Orthopaedic Surgery and Traumatology, Charité Berlin, University Hospital, Berlin, Germany

**Keywords:** White blood cell, Shoulder arthroplasty, Aspiration, Sensitivity, Specificity, Diagnostic test, Leukocyte count

## Abstract

**Background:**

Limited data exist regarding the diagnostic accuracy of synovial fluid leukocyte count (SFLC) in diagnosing periprosthetic shoulder infection (PSI). The main objective of this study was to determine the diagnostic value of leukocyte count at a common threshold of 3 cells/nL and the optimal cut-off value.

**Methods:**

Patients who underwent shoulder arthroplasty revision surgery and aspiration with SFLC between 2012 and 2023 were retrospectively included. The International Consensus Meeting 2018 definition was used to characterize infection status for SFLC threshold and synovial fluid neutrophil percentage (SFNP). Sensitivity and specificity were presented using cross tabulation. The area under the curve was calculated, and the optimal cut-off was determined using maximized Youden Index.

**Results:**

35 cases with an average age of 71 years (43% male) were included in our study. At a threshold of 3 cells/nL, SFLC showed a sensitivity of 70% and specificity of 83%. The corresponding positive and negative predictive values (PPV and NPV) were 89% and 59%, respectively. We found the optimal cut-off for our cohort at 4.7 cells/nL, increasing specificity to 92% while maintaining sensitivity at 70% (PPV = 94%, NPV = 61%). SFNP at a cut-off of 80% demonstrated 50% sensitivity and 91% specificity, with corresponding PPV and NPV of 92% and 48%, respectively. The optimum threshold for SFNP was 54%, which had a sensitivity of 77% and a specificity of 64%, as well as a PPV of 81%, and NPV of 58%. The area under the curve was 0.72 for SFLC and 0.74 for SFNP. Sonication detected pathogens in 63% of cases, while 57% of all cases showed positive tissue cultures and 43% positive aspirate cultures. Especially, the most frequently found microorganism, *Cutibacterium acnes*, was detected less often in aspirate culture.

**Conclusions:**

SFLC shows good specificity but moderate sensitivity for diagnosing PSI when using a threshold of 4.7 cells/nL. Therefore, it can serve as a confirmatory test for diagnosing PSI but not for ruling out infection.

Periprosthetic infection is a severe complication in arthroplasty, which often requires an extensive 1- or 2-stage revision that is accompanied by enormous burden for the patient.^12^ Therefore, an accurate diagnosis must be made to justify the treatment. The periprosthetic shoulder infection (PSI) has shown to be a diagnostic challenge, because of the high share of the low-virulence pathogen *Cutibacterium acnes* among all cases.^7^ It generates only a minor immune reaction and can even be found in the primary arthroplasty without causing significant inflammation.^9^^,^^19^ Therefore, typical signs of inflammation are rare and serum markers are not reliably altered.^2^^,^^3^^,^^11^^,^^18^ To specify periprosthetic joint infection (PJI), several expert associations have developed different diagnostic criteria, which include laboratory values like serum C-reactive protein, erythrocyte sedimentation rate, or synovial fluid leukocyte count (SFLC).

However, the recommended cut-off values for suspicious SFLC differ between 2 and 3 cells/nL^5^^,^^6^^,^^10^^,^^13^ and are mostly defined by data from hip or knee PJI, which are more likely caused by pathogens of higher virulence.^1^^,^^15^ Data on SFLC specifically in PSI is rare and based on only small case numbers.^11^^,^^17^ Additionally, deviating information about the diagnostic accuracy is available, and the comparison between other studies is complicated due to different infection classifications systems used. Therefore, this study aims to analyze the diagnostic value and optimum cut-off of SFLC in diagnosing PSI.

## Materials and methods

The ethical approval for this study was given prior to study initiation (EA4/040/14). We performed a retrospective cohort study of consecutively chosen patients who underwent synovial fluid sampling and revision shoulder arthroplasty between January 2012 and June 2023 in our institution.

Only patients who fulfilled both of the following criteria were included: 1) History of shoulder arthroplasty before synovial fluid sampling (primary or revision, unilateral or bilateral, hemi arthroplasty, anatomic total shoulder arthroplasty, or reverse total shoulder arthroplasty); 2) revision shoulder arthroplasty performed after synovial sampling. Reasons for exclusion were insufficient data for analysis. Information on C-reactive protein (CRP) was not available for one patient case, and the synovial fluid neutrophil percentage (SFNP) was not determined in two cases. Therefore, these cases were not included in the analysis of the missing parameter. Further exclusion criteria were less than three tissue specimens taken or major ipsilateral shoulder surgery within 6 weeks before synovial fluid testing.

Synovial aspirations were taken either intraoperatively or in an outpatient setting, strictly following aseptic standards. A sterile cannula was used either through an anterior or dorsal approach to aspirate a minimum volume of 1 ml synovial fluid. Aspirated fluid was never diluted and was transferred to a sterile test tube for leukocyte calculation and to a blood culture bottle for microbiologic testing. Blood culture bottles were incubated for 14 days. Furthermore, the proportion of polymorphonuclear cells in aspiration was calculated.

The International Consensus Meeting (ICM) definition was used as standard diagnostic criteria to diagnose PSI.^6^ In addition, the data were also classified by the European Bone & Joint Infection Society (EBJIS) definition of PJI from 2021 to improve comparability with other studies.^10^ For the analysis of the diagnostic utility of SFLC and SFNP in patients, the definitive, probable, and possible infection groups were combined and defined as infection group, which corresponds to likely or confirmed infection in EBJIS. Patients in the infection unlikely group within these definitions were defined as noninfection group. The infection type and host characteristics were categorized by McPherson classification.^4^

According to the standard PJI protocol at our institution, at least 3 periprosthetic tissue cultures from various suspicious surgical sites, at least one specimen for histopathologic analysis and retrieved implants for sonication analysis, were obtained in every patient at the time of revision surgery. Specimen for microbiological analysis were collected with a new sterile instrument each time, were placed directly into sterile containers without touching by hand, and sent immediately with retrieved implants to our microbiology laboratory for further analysis within 1 hour after surgery. The microbiologic specimen as well as sonication fluid cultures are plated onto aerobic and anaerobic sheep blood agar plates and incubated for 14 days. Sonication was performed as previously described.^14^

### Statistical analysis

IBM SPSS Statistics 29 (IBM Corp., Armonk, NY, USA) was used for statistical analysis. Patient characteristics are reported as mean and standard deviation SD. Testing for normal distribution was performed by Shapiro–Wilk test. Comparison between the infected and noninfected group were executed using Mann–Whitney U test, because all relevant variables were independent and non-normally distributed. The statistical significance was considered *P* < .05. The diagnostic values of sensitivity, specificity positive predictive value (PPV), and negative predictive value (NPV) were calculated using cross tabulation and receiver operating characteristics (ROC). The optimum cut-off was found by Youden-Index.

## Results

### Population characteristics

Following our inclusion and exclusion criteria, 35 shoulders in 33 patients were included in the analysis. The mean age was 71 years, ranging between 54 and 87 years and 43% being males. Classified by ICM-18, 8 (23%) cases were considered definite infection, 5 (14%) probable infection, and 10 (29%) possible infection, which resulted in 23 cases being categorized as infected and in the remaining 12 (34%) cases, an infection was classified as unlikely, while the EBJIS definition classified 25 cases as infected. According to McPherson, no cases of acute infection occurred, only one patient in each group had a significantly compromised status and the local extremity conditions were either moderately compromised (71%) or uncompromised (29%).

The reasons for surgery in aseptic patients were prosthetic loosening (7/12), clinically suspected infection, not confirmed according to ICM-18 (2/12), periprosthetic fracture (1/12), and dislocation (2/12). A detailed overview of case characteristics specified by their subgroup is given in Table I.

**Table I:** Case characteristics categorized by ICM.

	Infected (n = 23)	Not infected (n = 12)
Age in years	70.8 ± 9.5 (53.8-87.1)	72.1 ± 6.9 (59.3-80.6)
Males	11 (47.8%)	4 (33.3%)
Number of previous revisions		
0	16 (69.6%)	7 (58.3%)
1	4 (17.4%)	4 (33.3%)
2	1 (4.3%)	1 (8.3%)
≥ 3	2 (8.7%)	0 (0%)
Type of arthroplasty		
Anatomic	8 (34.8%)	1 (8.3%)
Reverse	15 (65.2%)	11 (91.7%)
Number of tissue samples	5.5 ± 1.2 (3-8)	5.3 ± 1.1 (4-8)
Aspiration during antibiotic treatment	3 (13.0%)	0 (0%)
McPherson		
I	0 (0%)	na
II	8 (34.8%)	na
III	15 (65.2%)	na
A	11 (47.8%)	4 (33.3%)
B	11 (47.8%)	7 (58.3%)
C	1 (4.3%)	1 (8.3%)
1	6 (26.1%)	4 (33.3%)
2	17 (73.9%)	8 (66.7%)
3	0 (0%)	0 (0%)

*ICM*, International Consensus Meeting.

McPherson: I = early postoperative infection (less than 4 weeks postoperative), II, hematogenous infection (less than 4 weeks duration), III, late chronic infection (more than 4 weeks duration), A = host status uncompromised, B = host status compromised, C = host status significantly compromised, 1 = local extremity uncompromised, 2 = local extremity compromised, 3 = local extremity significantly compromised; na = not applicable.

### Diagnostic accuracy of SFLC

The mean SFLC was significantly higher in infected group compared to noninfected group (73.9/nL (SD = 114.0) vs. 3.4/nL (SD = 6.0); *P* = .03). A SFLC cutoff of 3/nL showed a sensitivity of 70% and a specificity of 83%, a PPV of 89%, a NPV of 59%, and an accuracy of 74%, if ICM criteria were used. According to the ROC analysis, an optimum SFLC threshold of 4.7/nL had a sensitivity of 70% and a specificity of 92%, a PPV of 94%, a NPV of 61%, and an accuracy of 77% for detection of a shoulder PJI. The area under curve (AUC) was 0.72, and the ROC analysis had a significance level of *P* = .03, if ICM criteria were used. Using the EBJIS definition, a cutoff of 3/nL demonstrated a sensitivity of 64% and a specificity of 80% (PPV = 89%; NPV = 47%; accuracy = 69%). The ROC analysis of cases classified by EBJIS showed an AUC of 0.81 and had a significance level of *P* = .01. It produced two optimum thresholds that were equivalent according to the Youden-Index. These are 1.5 leukocytes/nL (sensitivity = 80%; specificity = 80%; PPV = 91%; NPV = 62%; accuracy = 80%) and 6.9 leukocytes/nL (sensitivity = 60%; specificity = 100%; PPV = 100%; NPV = 50%; accuracy = 71%). Detailed information about sensitivity and specificity at various thresholds and judged by different classification scores are provided in Table II.

**Table II:** Sensitivity and specificity of SFLC by classification system.

Threshold in Leukocytes/nL	0.5	1.1	1.5	2.5	3.8	4.7	5.7	6.9	12.5	56.3
ICM										
Sensitivity	0.87	0.74	0.70	0.70	0.70	0.70	0.65	0.61	0.57	0.43
Specificity	0.17	0.25	0.50	0.75	0.83	0.92	0.92	0.92	0.92	1.00
EBJIS										
Sensitivity	0.88	0.84	0.80	0.68	0.64	0.64	0.60	0.60	0.56	0.40
Specificity	0.20	0.50	0.80	0.80	0.80	0.90	0.90	1.00	1.00	1.00

*ICM*, International Consensus Meeting; *EBJIS*, European Bone & Joint Infection Society; *SFLC*, synovial fluid leukocyte count.

Optimum threshold for each classification system marked in grey.

### Diagnostic accuracy of SFNP

The mean SFNP displayed a significant increase in the infected group (73%, SD = 28%) in contrast to the noninfected group (54%, SD = 20%), *P* = .02. Applying ICM criteria, a SFNP cutoff at 80% showed a sensitivity of 50% and a specificity of 91%, a PPV of 92% with a NPV of 48%, and an accuracy of 64%. ROC analysis suggested an optimal SFNP threshold of 54%, resulting in a sensitivity of 77% and a specificity of 64%, along with a PPV of 81%, an NPV of 58%, and an accuracy of 73% for identifying shoulder PJI. The AUC was 0.75, with a significance level of *P* = .02, using ICM criteria. When implementing the EBJIS definition, a cutoff of 80% displayed a sensitivity of 50% and a specificity of 100% (PPV = 100%; NPV = 43%; accuracy = 64%). ROC analysis based on EBJIS classification had an AUC of 0.83, *P* < .01. The optimal SFNP threshold identified was 68% (sensitivity = 75%; specificity = 100%; PPV = 100%; NPV = 60%; accuracy = 82%). More details are available in Table III.

**Table III:** Sensitivity and specificity of SFNP by classification system.

Threshold %	0.28	0.37	0.46	0.54	0.59	0.68	0.76	0.82	0.88	0.96
ICM										
Sensitivity	0.91	0.86	0.77	0.77	0.73	0.68	0.59	0.55	0.45	0.23
Specificity	0.00	0.27	0.36	0.73	0.73	0.73	0.73	0.91	0.91	1.00
EBJIS										
Sensitivity	0.96	0.83	0.79	0.79	0.75	0.75	0.67	0.54	0.46	0.21
Specificity	0.11	0.22	0.44	0.89	0.89	1.00	1.00	1.00	1.00	1.00

*ICM*, International Consensus Meeting; *EBJIS*, European Bone & Joint Infection Society; *SFNP*, synovial fluid neutrophil percentag.

Optimum threshold for each classification system marked in grey.

### Serum parameters and identified microorganisms

Defined by ICM, the mean serum-CRP in the noninfected group (n = 12) was 10.7 mg/l (SD = 16.3), whereas the infected group (n = 22) showed a mean CRP of 44.8 mg/l (SD = 71.4). However, the difference between both groups was not significant (*P* = .14).

**Figure 1:**
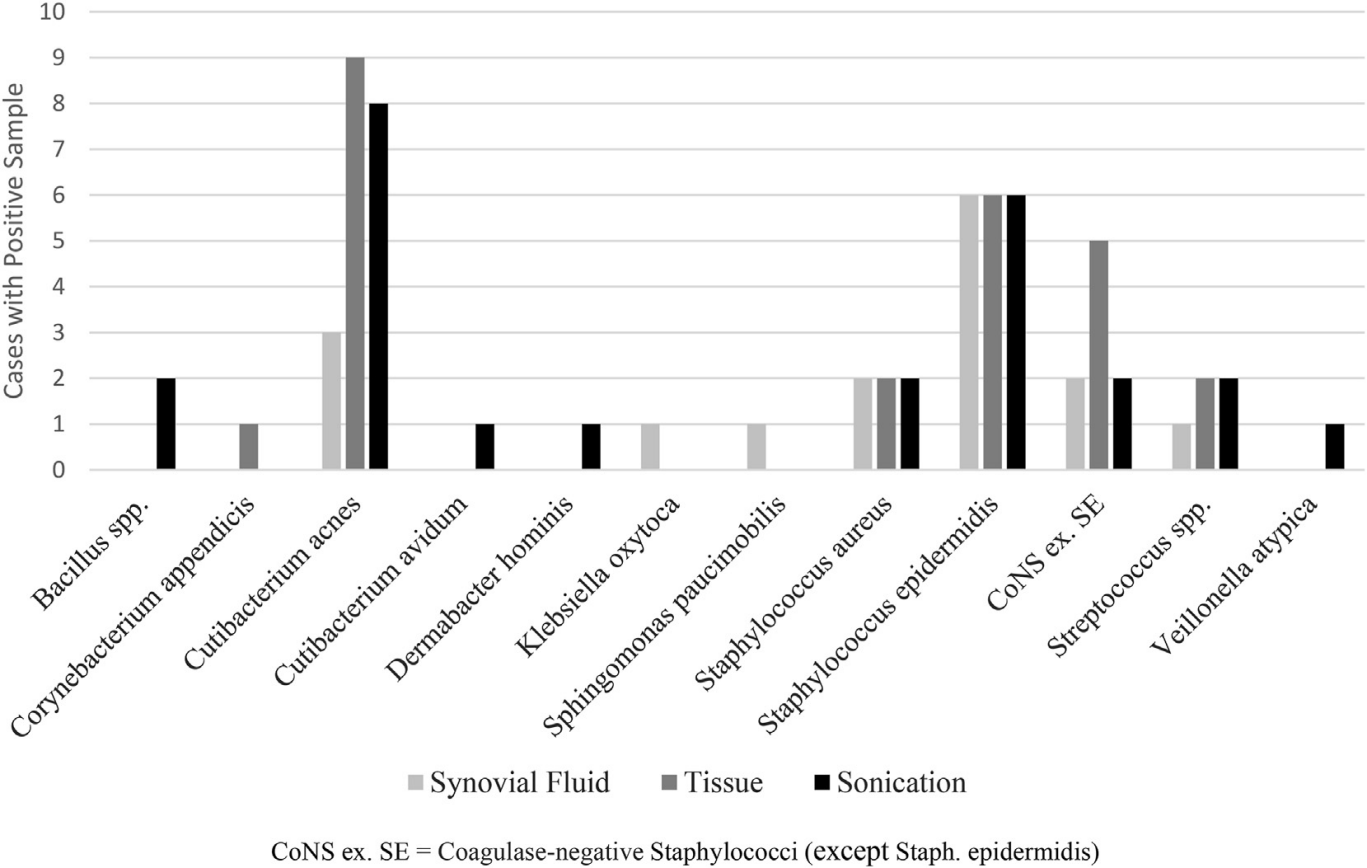
Cultivated bacteria by sampling method.

The microbiological assessment of the synovial fluid was not possible in 5 out of 35 cases (14%), because the amount of drained fluid was insufficient for both laboratory and microbiological testing. It found bacteria in 13 of 30 cases (43%), while 20 of all 35 cases (57%) had positive tissue samples, and sonication was able to identify bacteria in 22 of all 35 cases (63%). Compared to microbiological analysis of synovial fluid, tissue sampling found the same bacteria in 12 (40%) and sonication in 13 (43%) of these cases. The most frequently isolated microorganism was *Cutibacterium acnes*, which was cultivated more often by samples originated from tissue or sonication than from synovial fluid. Other common bacteria were *Staphylococcus epidermidis* followed by *Staphylococcus aureus* and other coagulase-negative staphylococci (except *Staph. epidermidis*). (Fig. 1).

## Discussion

Shoulder PJI presents a unique diagnostic challenge because of the low virulence of the most common causative microorganisms. The determination of the infection status in patients with a clinically failed shoulder arthroplasty is a key step in the treatment planning. Therefore, the identification of potential biomarkers for an accurate diagnosis of shoulder PJI is of importance. Although SFLC is a commonly used parameter to detect PJI especially in hip and knee, the literature in PSI is very scarce, and no threshold is defined. In our study, SFLC showed a good specificity but a moderate sensitivity for the diagnosis of PSI, when a threshold of 4.7 cells/nL is used. Likewise, SFNP had a mediocre sensitivity and good specificity at thresholds between 54% and 68% in our cohort.

Previous studies addressing the diagnostic accuracy of SFLC in PSI showed deviating results. A recently published study by Streck et al had found an optimum diagnostic accuracy of SFLC of 87% sensitivity and 88% specificity at >2.8 cells/nL.^17^ This difference can be explained by the diagnostic criteria used by the authors (Guidelines by the Infectious Diseases Society of America).^12^ Mederake et al used the MSIS 2014 definition and showed a sensitivity of 20% and specificity of 82% at a leukocyte threshold of 1.1/nL.^11^ Our data, calculated using the ICM definition and the same threshold, suggests a sensitivity of 74% and specificity of 25%, which is very different from the comparative study. However, Mederake et al made the SFLC analysis dependent on the synovial fluid remaining after microbiology sampling and therefore included only 16 patients, which might have introduced bias by selection for SFLC testing.

Our analysis shows that the diagnostic accuracy depends on the diagnostic criteria used. The sensitivity differed by 6% and specificity by 2% between ICM and EBJIS criteria at 4.7 leukocytes/nL. Moreover, the overall number of infected cases differed slightly between 23 for ICM and 25 for EBJIS. This is the consequence of EBJIS considering cases as likely infected more easily. Precisely, the thresholds for likely infection are smaller in EBJIS, ie, the proportion of polymorphonuclear leukocytes is suspicious at more than 65%, whereas ICM requires more than 80%. Our data implies that a cutoff of 68% neutrophils already results in high specificity, whereas higher thresholds would only decrease sensitivity when utilizing EBJIS criteria. Considering the ICM, a cutoff of 54% presents both moderate sensitivity and specificity.

Our results show that the threshold of 3 leukocytes/nL, which is commonly used in periprosthetic infection definitions, has insufficient sensitivity and specificity to reliably diagnose PSI. An increased threshold of 4.7/nL, as shown in our study, might improve specificity, but further research is needed to verify this finding, especially since comparable studies reported deviating results. Despite that, clinicians should not solely rely on synovial diagnostic, including SFLC, SFNP, and aspiration cultures, since intraoperative tissue and sonication samples had a superior capability of finding microorganisms.

Our study faced several limitations. First, the number of included parameters within the diagnostic scores varied between the individual cases. We were not able to include variables like synovial alpha-defensin or nuclear imaging because these are not determined by our laboratory in standard care. Second, we only investigated SFLC and SFNP as single diagnostic markers, but in diagnostic scores, it is combined with multiple other variables, which might change its ideal threshold, as a previous study showed.^16^ As our McPherson categorization demonstrates, none of our cases had an early postoperative infection. For this reason, our results are only applicable to chronic PSIs, because early infections are expected to require higher thresholds.^8^ Furthermore, the synovial sampling was only performed if the clinician saw a diagnostic benefit, which could have caused selection bias.

## Conclusions

SFLC showed a good specificity but a moderate sensitivity for the diagnosis of chronic PSI, when a threshold of 4.7 cells/nL is used. Thus, it may be used as a confirmatory test in the diagnosis of PSI but not to rule out infection.

## Disclaimers:

Funding: No funds, grants, or other support were received for the purpose of this study.

Conflicts of interest: The authors, their immediate families, and any research foundation with which they are affiliated have not received any financial payments or other benefits from any commercial entity related to the subject of this article.
